# Cabergoline-induced prolactinoma treatment and subsequent estrogen-driven vascular smooth muscle tumorigenesis in a middle-aged female: A case report

**DOI:** 10.1016/j.ijscr.2025.111786

**Published:** 2025-08-15

**Authors:** K. Cintrón-Cartagena, G. Christian-Colón, R. Ortiz-Figueroa, F. Vélez-Alvarado, I. Torres-Milán, G. Ruiz-Deyá

**Affiliations:** a Urology Department, Saint Luke's Episcopal Hospital, Ponce, Puerto Rico; b Ponce Health Sciences University, School of Medicine, Ponce, Puerto Rico

**Keywords:** Renal angioleiomyoma, Incidentaloma, Puerto Rican, Hispanic, Case report

## Abstract

**Introduction and importance:**

Renal angioleiomyoma is an exceptionally rare benign tumor of smooth muscle origin, often discovered incidentally and easily mistaken for other differential diagnoses on imaging.

**Case presentation:**

We report the case of a 34-year-old Puerto Rican woman with a history of prolactinoma treated with cabergoline, who was found to have a right renal-adrenal mass on imaging. She underwent successful laparoscopic resection. Histopathological analysis confirmed angioleiomyoma based on spindle cell morphology, positive immune markers, and a low Ki-67 index.

**Discussion:**

Notably, this case suggests a potential hormonal etiology, with rebound estrogen elevation post-prolactinoma treatment possibly stimulating angiogenesis and smooth muscle proliferation via VEGF pathways. The absence of trauma, venous stasis, or genetic abnormalities supports a hormonally driven mechanism, meriting further exploration in hormonally active female patients.

**Conclusion:**

This case highlights the need for urologists and research fellows to consider hormonal history when evaluating incidental renal masses. Hormonal rebound following endocrine therapy may play an underrecognized role in tumor pathogenesis. Surgical excision remains the definitive treatment for diagnostic clarification and curative intent.

## Highlights

• Incidental renal adrenal mass found in patient with long-standing history of prolactinoma
• Mass was removed via nephrectomy and pathology confirmed angioleiomyoma.
• Suspected formation of mass from estrogen upregulation due to prolactinoma medical management
• First Puerto Rican diagnosed with this condition

## 1 Introduction

Leiomyoma is a benign tumor originating from leiomyocytes—smooth muscle cells derived from mesenchymal tissue [1]. Leiomyomas are classified into subtypes based on their etiology, including angioleiomyomas and piloleiomyomas. Piloleiomyomas arise from the arrector pili muscles, typically manifesting as painful cutaneous lesions widely distributed on the skin [2]. Genital leiomyomas, occurring in areas such as the scrotum and vulva, often present as pruritic lesions and are generally less painful [3]. Among gynecological manifestations, uterine leiomyoma represents a common subtype [4].

Angioleiomyoma is the most prevalent subtype of leiomyoma, originating from vascular smooth muscle, specifically the tunica media [5]. There is a noted higher incidence in females, with peak presentation commonly occurring during the third decade of life [6]. Angioleiomyomas account for approximately 5 % of all benign soft tissue neoplasms [7]. Histologically, angioleiomyomas are classified into three types—capillary, cavernous, and venous—based on the predominant blood vessel component present [7]. The capillary type is most frequently observed in females, whereas cavernous and venous types are more common among males [7]. Renal angioleiomyoma, however, is exceedingly rare, constituting less than 1 % of primary renal tumors [8].

Diagnosing renal angioleiomyoma poses significant challenges due to its deep anatomical location and uncommon occurrence [8]. Advances in contrast-enhanced imaging techniques have improved tumor detection and characterization, aiding differentiation from other renal masses such as angiomyolipomas and renal cell carcinomas [9]. These imaging modalities provide detailed visualization of the tumor's vascular structure; nonetheless, definitive diagnosis depends on histopathological confirmation via biopsy [9].

Renal leiomyomas frequently are incidental findings but may manifest clinically with abdominal or flank pain, palpable masses, or hematuria in approximately 20 % of symptomatic patients [10]. In this report, we present a case involving a female patient with an incidentally identified right renal mass diagnosed histologically as angioleiomyoma. To our knowledge, this is the first documented case of renal angioleiomyoma in a Puerto Rican patient. This case report adheres to the SCARE 2025 criteria [11].

## 2 Case presentation

**Fig. 1:**
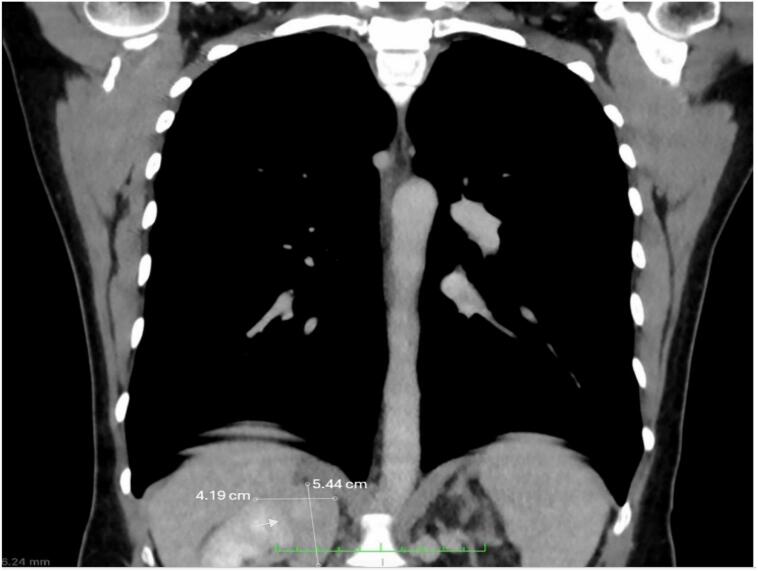
Coronal view of chest CT scan showing an enhancing mass measuring 5.4 × 4.1 cm. The mass is said to project along the right suprarenal aspect or exophytic from the superior right renal cortex.

A 34-year-old Puerto Rican female was referred to the Urology Clinic at a local hospital following the incidental discovery of a right renal-adrenal mass (Fig. 1). She reported nocturia, weak urinary stream, urinary frequency, and persistent, non-radiating right flank pain. Her medical history was significant for a prolactinoma associated with a pituitary microadenoma diagnosed 12 years prior, treated successfully with cabergoline; serum prolactin levels had normalized less than one year before her current presentation. Family history was negative for renal tumors, although her nephew had documented pituitary microadenoma. Her gynecological history was notable for infertility. She denied tobacco, alcohol, illicit drug use, or relevant environmental exposures. Additionally, there was no personal history or ultrasonographic evidence of uterine fibroids.

**Fig. 2:**
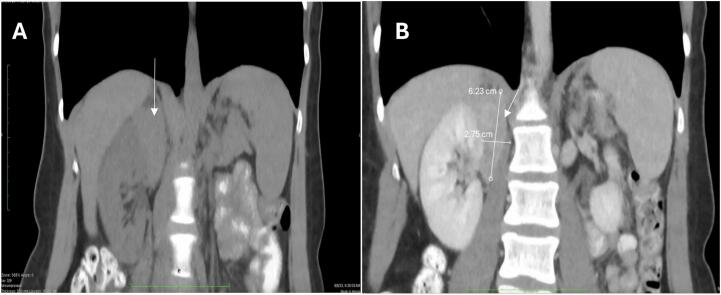
(A) and (B) Abdominal CT scan without and with contrast showing an enhancing mass measuring 6.2 × 2.7 × 5.5 cm. The mass is said to originate from the right kidney or from the tip of the medial limb of the adrenal gland.

Abdominal computed tomography (CT) imaging demonstrated a suprarenal mass on the right side (Fig. 1, Fig. 2). Complete blood count, electrolyte panel, and biochemical profiles were within normal limits. Renal function was preserved, evidenced by normal serum creatinine levels and glomerular filtration rates. Endocrine evaluations, including serum aldosterone, plasma metanephrine, and normetanephrine levels, were within reference ranges.

The patient underwent laparoscopic resection of the right retroperitoneal mass. Pneumoperitoneum was achieved using the Veress needle technique, and laparoscopic ports were strategically placed in a triangulated configuration. The colon was mobilized medially to expose the retroperitoneum adequately, facilitating careful dissection of the renal hilum to isolate and secure the renal artery, vein, and ureter. The mass was observed extending between the renal vessels; nonetheless, precise vessel identification and clamping were successfully accomplished without complications.

Intraoperative exploration revealed that the mass originated from the kidney and was inseparable from the adrenal gland. Thus, partial nephrectomy and adrenalectomy were performed, removing both the mass and the adrenal gland. The patient tolerated the procedure well, experiencing an uneventful postoperative recovery, and was discharged home with arrangements for outpatient follow-up to discuss histopathological findings.

**Figs. 3:**
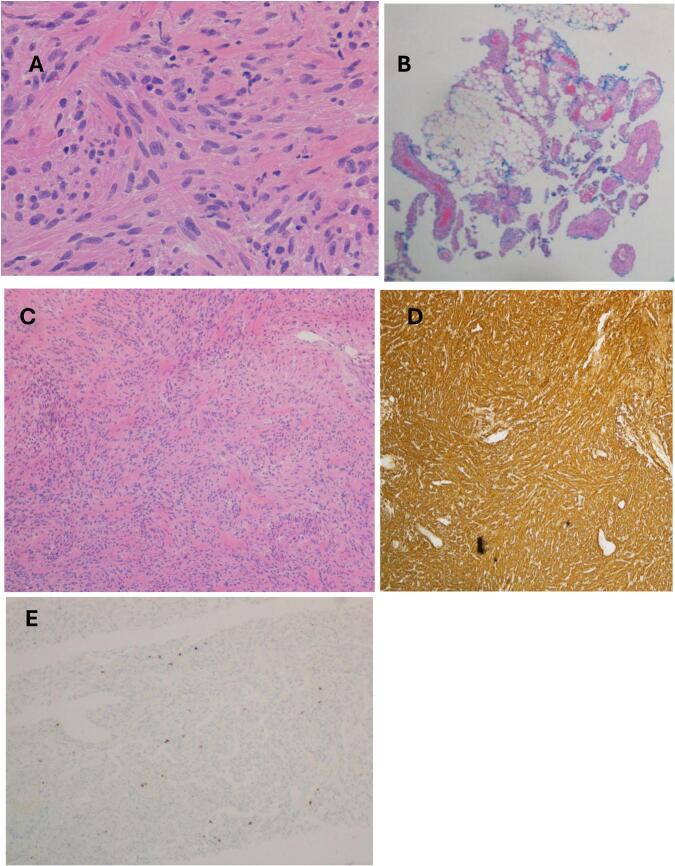
Pathology slides from renal mass from hematoxylin and eosin (H&E) and immune markers. (A) H&E Magnification ×40, (B) H&E Magnification ×4, (C) H&E Magnification ×10, (D) an immunohistochemical stain for Caldesmon shows strong and diffuse cytoplasmic staining, and (E) an immunohistochemical stain for Ki-67 shows nuclear staining with a low proliferation index.

Histopathological analysis encompassed examination of three specimens obtained intraoperatively: Right Adrenal gland, Right Kidney Biopsy, and Retroperitoneal Mass. Histologically, the mass was consistent with a benign smooth muscle tumor, characterized by spindle-shaped cells with eosinophilic cytoplasm, minimal mitotic activity, and absent cytologic atypia. Immunohistochemistry demonstrated positivity for smooth muscle markers, including smooth muscle actin (SMA), caldesmon, and desmin, with a low nuclear Ki-67 proliferative index (Fig. 3). This immunoprofile supported a diagnosis of a smooth muscle neoplasm with low proliferative potential, consistent with angioleiomyoma (Table 1).

**Table 1:** Microscopic diagnosis summary.

Specimen	Procedure	Diagnosis	Comments
Right adrenal	Excision	Angioleiomyoma	Negative for malignancy
Right kidney	Biopsy	Benign renal tissue identified	
Retroperitoneal mass	Resection	Angioleiomyoma; Benign renal tissue identified	Negative for malignancy

**Fig. 4:**
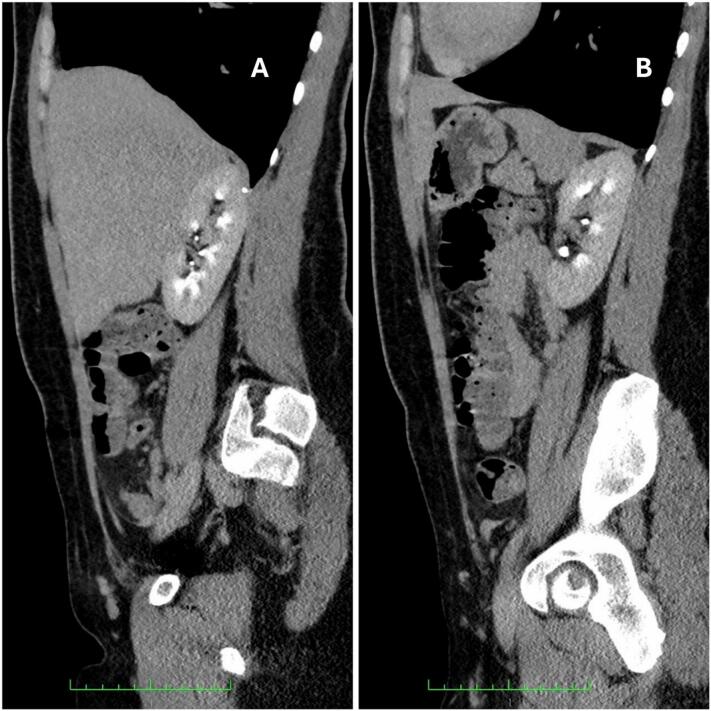
Abdominopelvic CT scan, sagittal views. (A) Shows the patient's right side of the body without any enhancing renal lesions. (B) Is only remarkable for an enlarged, nonspecific, para-aortic lymph node in the patient's left side of the body. This left para-aortic lymph node enlargement remains under close observation, with follow-up imaging planned to monitor any changes in size or characteristics.

Postoperative urological follow-up showed stable laboratory parameters. Renal ultrasound revealed irregular cortical thinning in the lateral interpolar region of the right kidney, consistent with postoperative structural changes. Follow-up abdominal CT imaging demonstrated no recurrence of disease (Fig. 4, sagittal view).

## 3 Discussion

This case of renal angioleiomyoma is notable not only for its rarity but also for the suggested hormonal etiology, an association scarcely reported in the literature. The distinctive aspect of this case lies in the proposed hormonal mechanism involving prolactin and estrogen. Prolactin typically exerts inhibitory control over gonadotropin-releasing hormone (GnRH), reducing estrogen synthesis through decreased secretion of luteinizing hormone (LH) and follicle-stimulating hormone (FSH). Successful prolactinoma treatment with cabergoline in our patient likely reversed chronic estrogen suppression, resulting in a potential rebound elevation of estrogen levels. Estrogen resurgence may facilitate angiogenesis and smooth muscle proliferation via vascular endothelial growth factor (VEGF)-mediated pathways [[14], [15], [16], [17]].

Estrogen significantly influences prolactin activity by upregulating prolactin receptors and enhancing its mitogenic effects. Estrogen's crucial role was studied in prolactinoma pathogenesis, emphasizing its broader implications in prolactin-responsive tissues [16,17]. In addition, estrogen's interaction with proangiogenic growth factors in prolactinomas suggests similar mechanisms could be active in angioleiomyoma pathogenesis [17]. This interaction is consistent with known estrogen-mediated VEGF upregulation, extensively documented in hormonally responsive smooth muscle tumors, notably uterine leiomyomas [[16], [17], [18]]. Given the shared smooth muscle and vascular characteristics, renal angioleiomyomas likely may respond similarly to estrogenic stimulation.

Alternative etiologies such as trauma or venous stasis were absent in this patient's clinical history, further solidifying the hormonal hypothesis. Nevertheless, this is merely theorical. Additionally, although certain angioleiomyomas correlate with specific chromosomal abnormalities [19], genetic testing in this patient did not reveal pathogenic alterations, thereby supporting an external factor rather than a genetic mechanism.

This report underscores a potentially novel pathogenic mechanism involving estrogen rebound following prolactinoma treatment, meriting further investigation among premenopausal women with hormonal disorders affecting estrogen regulation. Management typically involves surgical intervention, with laparoscopic excision proving curative in this case [20].

Limitations include the absence of longitudinal hormonal assessments, particularly estrogen levels, which would provide direct evidence of hormonal rebound. Also, despite negative genetic findings, epigenetic influences remain possible and warrant further research.

## 4 Conclusion

Renal angioleiomyoma is a rare benign tumor of smooth muscle origin, often discovered incidentally and posing diagnostic challenges due to its resemblance to other renal neoplasms. This case underscores a potentially underrecognized hormonal mechanism involving estrogen rebound after successful prolactinoma treatment, providing valuable insights into tumor pathogenesis.

Key Takeaways:

• Clinicians should be alert to hormonal rebound effects, particularly estrogen normalization, in patients undergoing long-term therapy for prolactinomas.
• When incidental renal masses are detected in hormonally treated women, benign vascular neoplasms such as angioleiomyoma should be included in differential diagnoses.
• Close monitoring for potential neoplastic changes is advisable following abrupt hormonal normalization, especially regarding estrogen levels.
• This case highlights the diagnostic complexity in distinguishing benign renal masses from malignant lesions, emphasizing the need for careful consideration and thorough investigation.
• Awareness and consideration of rare entities like angioleiomyoma can facilitate more accurate diagnosis and avoid overtreatment.
• Surgical management remains effective for indeterminate renal masses when malignancy cannot be definitively excluded by imaging alone.

By elucidating the importance of endocrine history in diagnostic evaluation, this report contributes significantly to the limited literature on renal angioleiomyoma and highlights crucial considerations in patient management.

## Author contribution

Kenneth Cintron-Cartagena, MD

Contributions:

- Study concept, data collection

Gustavo Christian-Colón, BS

Contributions:

- Data interpretation, writing the paper

Rodrigo Figueroa-Ortiz, BS

Contributions:

- Data analysis, writing the paper

Frances Vélez-Alvarado, BS

Contributions:

- Writing the paper

Isabel Torres-Milán

- Writing the paper

Gilberto Ruiz-Deyá, MD

- Study concept, Study design

## Consent

Written informed consent was obtained from the patient for publication and any accompanying images. A copy of the written consent is available for review by the Editor-in-Chief of this journal on request.

## Ethical approval

Approved by IRB at local institution. IRB Protocol Number: 2411224026.

Institution Name: Ponce Health Sciences University.

## Guarantor

Kenneth Cintron Cartagena, MD.

## Research registration number

Case report is not a “First in Man” study.

## Funding

No funding used to write this case report.

## Conflict of interest statement

No conflict of interests to report.
